# Genome-Wide Analysis of Alternative Splicing Provides Insights Into Stress Response of the Pacific White Shrimp *Litopenaeus vanname*

**DOI:** 10.3389/fgene.2019.00845

**Published:** 2019-09-12

**Authors:** Xiaoxi Zhang, Jianbo Yuan, Xiaojun Zhang, Chengzhang Liu, Jianhai Xiang, Fuhua Li

**Affiliations:** ^1^Key Laboratory of Experimental Marine Biology, Institute of Oceanology, Chinese Academy of Sciences, Qingdao, China; ^2^Laboratory for Marine Biology and Biotechnology, Qingdao National Laboratory for Marine Science and Technology, Institute of Oceanology Chinese Academy of Sciences, Qingdao, China; ^3^University of Chinese Academy of Sciences, Beijing, China; ^4^Center for Ocean Mega-Sciences, Chinese Academy of Sciences, Qingdao, China

**Keywords:** alternative splicing (AS), stress response, *Litopenaeus vannamei*, RNA-seq, genome-wide identification

## Abstract

Alternative splicing (AS) can enhance transcript diversity dramatically and play an important role in stress adaptation. Limited researches of AS have been reported in the Pacific white shrimp (*Litopenaeus vannamei*), which is an important aquaculture species in the world. Here, we performed a genome-wide identification of AS events in *L. vannamei* based on eight transcriptomes. We identified 38,781 AS events in the shrimp genome, and some of them were validated by polymerase chain reaction experiments. These AS events correspond to 9,209 genes, accounting for 36% of protein-coding genes in the shrimp genome. The number of AS events increased after virus or bacteria infection and low salinity stress. Type 1 AS genes (AS was initially activated) were mainly enriched in substance and energy metabolism, such as carbon metabolism and amino metabolism. However, type 2 AS genes (AS events changed) displayed specific enrichment under different stress challenges. Specifically, type 2 AS genes under biotic stresses were mainly enriched in the pathogenic pathway and immune network, and the AS genes under low salinity stress were significantly enriched for betalain biosynthesis. In summary, our study indicates that AS events are complex in shrimp and may be related to stress adaptation. These results will provide valuable resource for functional genomic studies on crustaceans.

## Introduction

Alternative splicing (AS) is an essential posttranscriptional regulatory mechanism in eukaryotic organisms that yield thousands of splice variants for one gene by using different splice sites (Huang et al., 2016). AS can not only determine the localization of the mature mRNA but also affect their translation efficiency (Petrillo et al., 2014). Nevertheless, AS may also produce premature termination codons (PTCs) because of the shifts of open-reading frame (ORF) in the mature mRNA sequence, thus regulating mRNA abundance and committing the transcripts to degradation *via* nonsense-mediated decay (NMD) (Maquat, 2004; Chang et al., 2007; Lareau et al., 2007). Thus, AS can modulate gene expression, transcriptome plasticity, and proteome diversity enormously (Lareau et al., 2004; Kelemen et al., 2013). AS can be classified into four types: exon skipping (ES), intron retention (IR), alternative 5’ splice site (A5SS), and alternative 3’ splice site (A3SS) (Black, 2003). A combination of these basic AS types can generate complex AS events (Walters et al., 2013). ES is the most common AS type and IR is the least AS event occurring in animals (Modrek and Lee, 2002; Sultan et al., 2008; Wang et al., 2008).

Genome-wide analysis of AS have been reported in several animal species based on expression sequence tags (ESTs) and RNA-sequencing. The AS events varied greatly within species. Approximately 95% of multi-exon genes in human (Pan et al., 2008; Wang et al., 2008), 31% in fruit fly (*Drosophila melanogaster*) (Graveley et al., 2011), more than 25% in nematode (*Caenorhabditis elegans*) (Ramani et al., 2011), 17% in zebrafish (*Danio rerio*) (Lu et al., 2010), and 16% in Pacific oyster (*Crassostrea gigas*) (Huang et al., 2016) are subjected to AS.

AS is usually involved in many physiological processes, including the response to biotic and abiotic stresses (Mastrangelo et al., 2012; Rodrigues et al., 2013; Gamazon and Stranger, 2014). Although large-scale AS events have also been characterized in some invertebrates (Lu et al., 2010; Graveley et al., 2011; Ramani et al., 2011; Huang et al., 2016), the study of AS regulation under stress was still relatively scarce in shrimp, or even in crustaceans. The Pacific white shrimp, *Litopenaeus vannamei*, is one of the most economically important marine aquaculture species and also the most produced shrimp species in the world (FAO, 2014). In consideration of the intensification of shrimp farming that was accompanied by infection of pathogens, environmental stress, and so on, investigating AS regulation will broaden our understanding of response mechanisms of shrimp to many stressors.

To fill the gap in AS research on crustaceans, we first performed a genome-wide analysis of RNA-seq data to identify AS events in the shrimp genome. We found that the number of AS events apparently increased after white spot syndrome virus (WSSV) or bacteria infection and low salinity challenge. In addition, we found that the genes with differential AS were well associated with specific functional categories and the responses to various stresses. Our study will complement the gene database of shrimp and provide a valuable resource for future functional genomic studies of crustaceans.

## Materials and Methods

### RNA-seq Libraries

A total of 112 libraries belonging to eight transcriptomes of *L. vannamei* were collected. They came from 5 larval stages, including embryo, nauplius, zoea, mysis, and post-larvae (Wei et al., 2014); 8 molting stages, including the inter-molt (C), pre-molt (D0, D1, D2, D3, D4), and post-molt (P1 and P2) stages (Gao et al., 2015); 16 adult tissues, including antennal gland, brain, hemocyte, epidermis, eyestalk, gill, hepatopancreas, heart, intestine, abdominal muscle, lymphoid organ, ovary, stomach, testis, thoracic ganglion, abdominal ganglion (Zhang et al., 2018); 3 biotic stressors, including WSSV, *Vibrio parahaemolyticus*, and *Staphylococcus aureus* infection samples (hemocyte, hepatopancreas, and lymphoid organ were collected from the infection groups after 6 h) (SRX3886087, SRX3886088, SRX3886089, SRX3886090, SRX3556303, SRX3556304, SRX3556305, SRX3556306, SRX3556307, SRX3556308, SRX3556309, SRX3556310, SRX3556311, SRX3556312, SRX3556291, SRX3556292, SRX3556278, SRX3556279, SRX3556280, SRX3556257).

### AS Event Identification

The low-quality reads were removed by Trimmomatic (Bolger et al., 2014), and the trimmed data from each sample were mapped to the shrimp genome (Zhang et al., 2019) using HISAT2 (v2.1.0) with default parameters (Kim et al., 2015). The SAM files generated by HISAT2 were converted to BAM files and sorted using SAMtools v.1.3 (Li et al., 2009). Next, transcripts were reference-based assembled individually for each library using StringTie (v1.3.4) with default settings (Pertea et al., 2015). To identify all AS events of shrimp genome, assembled transcripts of all samples were merged using StringTie-merge. The transcript abundances were further estimated with “-eB” parameter, and gene expression levels were assessed using FPKM values. Then, AS identification were performed using ASTALAVISTA algorithm (v4.0) (Foissac and Sammeth, 2007). Various types of AS events, including exon skipping (ES, AS code: 1-2^, 0), intron retention (IR, AS code: 1^2-, 0), alternative 3’ splice sites (A3SS, AS code: 1-,2-), alternative 5’ splice site (A5SS, AS code: 1^,2^), and mutually exclusive exon (MXE, AS code: 0,1-2^,3-4^), were analyzed as previously defined (Sammeth et al., 2008). The common or specific AS genes between different libraries were pictured by the online tool Venn diagram (http://bioinformatics.psb.ugent.be/webtools/Venn/). The reads densities and distribution were visualized by the Integrated Genome Browser (Nicol et al., 2009).

### PCR Validation of AS Events

To validate predicted AS events, the selected ES, RI, A5SS, and A3SS events were validated by polymerase chain reaction (PCR) using a set of primers (**Supplementary Table 1**) that were designed based on each AS event. For each sample, 1.5 μg total RNA was reverse transcribed into first-strand cDNA using a PrimeScript RT reagent kit (TaKaRa). PCR was performed in a 25-μl reaction system using Premix Ex Taq™ Hot Start Version (TaKaRa), and the procedure was as follows: initial denaturation at 95°C for 1 min; 95°C for 10 s, 55°C for 30 s, and 72°C for 1 min for 35 cycles; and a final extension at 72°C for 5 min. The PCR products were visualized by 1.5% agarose gel electrophoresis.

### Characteristics of AS and Non-AS Genes

To elucidate the characteristics of AS and non-AS genes, gene length, exon number, exon length, and intron length were calculated using in-house python-script (https://github.com/XiaoziZhang/AS-events-of-Litopenaeus-vannamei). In addition, to test whether stress conditions induce any bias of splicing sites motif patterns, splicing site motifs of non-AS genes and AS genes under each condition were subtracted and compared with each other.

### Gene Ontology and KEGG Analysis

Gene Ontology (GO) and Kyoto Encyclopedia of Genes and Genomics (KEGG) annotation of AS genes were conducted by the OSGO tool of OmicShare (http://www.omicshare.com/tools). GO and KEGG enrichment analysis was performed using clusterProfiler (Yu et al., 2012), with a significant score p-value calculated under hypergeometric distribution.

## Results

### Large-Scale Identification of AS Events in *L. vannamei*

To investigate AS events in shrimp, more than 2,630 million reads (∼1.734 Tb) from 112 high-throughput RNA-seq libraries were collected. A total of 137,143 transcripts were assembled, and 38,781 AS events corresponding to 9,209 genes were identified, accounting for 39.5% of intron-containing genes (23,313) in the shrimp genome. This percentage is similar to that of the fruit fly (*D. melanogaster*, 31%) (Graveley et al., 2011), higher than the Pacific oyster (*C. gigas*, 16%) (Huang et al., 2016), zebrafish (*D. rerio*, 17%) (Lu et al., 2010), and nematode (*C. elegans*, 25%) (Ramani et al., 2011) but lower than that in human (*Homo sapiens*, 95%) (Pan et al., 2008; Wang et al., 2008). In total, there were 38,781 AS events distributed on 2,680 scaffolds, accounting for 57.2% of the shrimp genome. The number of AS events of top 20 scaffolds ranged from 147 to 565 (**Supplementary Figure 1**). The scaffold 2,331 and scaffold 976 had the largest number of AS events, 565 and 393 AS events, respectively.

### Identification and Validation of Different AS Types

**Figure 1:**
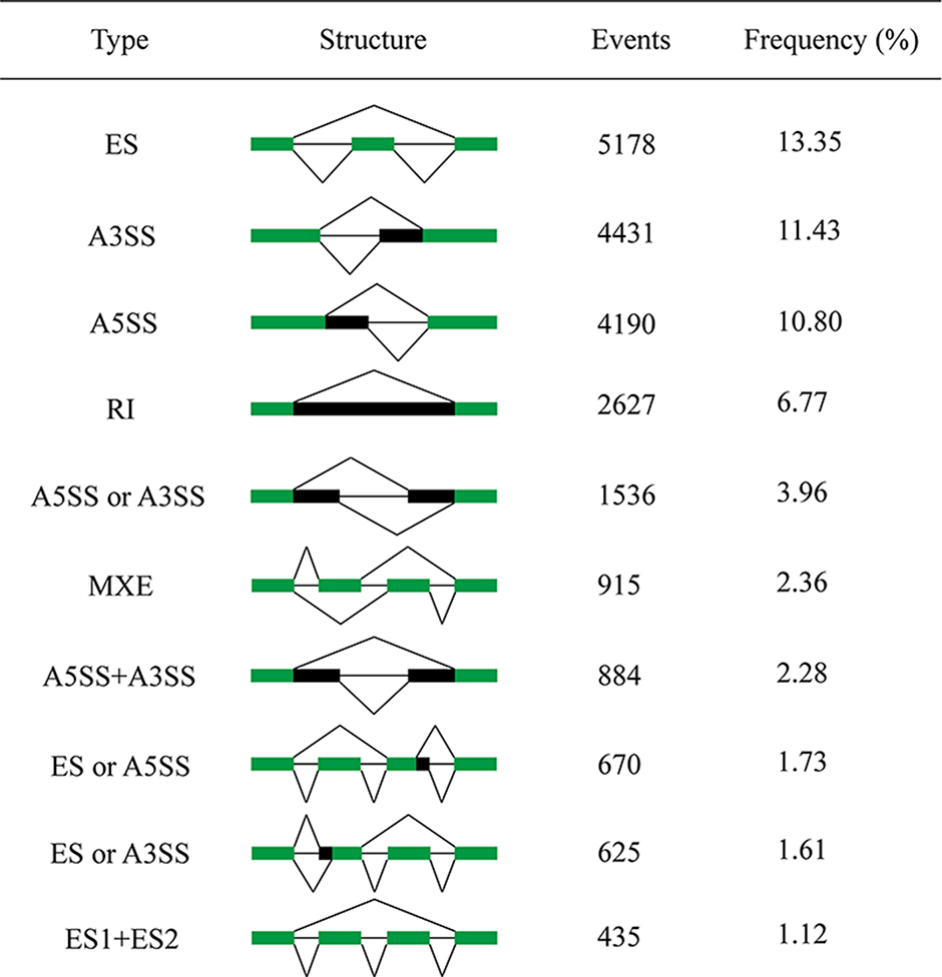
Top 10 most frequent types of AS in the predicted transcripts. The first column illustrates the description of AS event type, followed by its intron-exon structure, the raw number of events found in the sample, and their frequency. ES, exon skipping; A3SS, alternative 3’ splicing site; A5SS, alternative 5’ splicing site; RI, intron retention; MXE, mutually exclusive exons. The green blocks represent exons, and the black blocks represent introns.

We identified various types of AS events in the shrimp genome (**Figure 1**). ES was the most abundant AS type, representing 13.35% of the total AS events, followed by A3SS (11.43%), A5SS (10.8%), RI (6.77%), A5SS/A3SS (3.96%), MXE (2.36%), A5SS+A3SS (2.28%), ES/A5SS (1.73%), ES/A3SS (1.61%), and ES1+ES2 (1.12%). Other complex AS events were shown in **Supplementary Figure 2**. These results showed that AS type in shrimp was considerably extensive and complicated.

**Figure 2:**
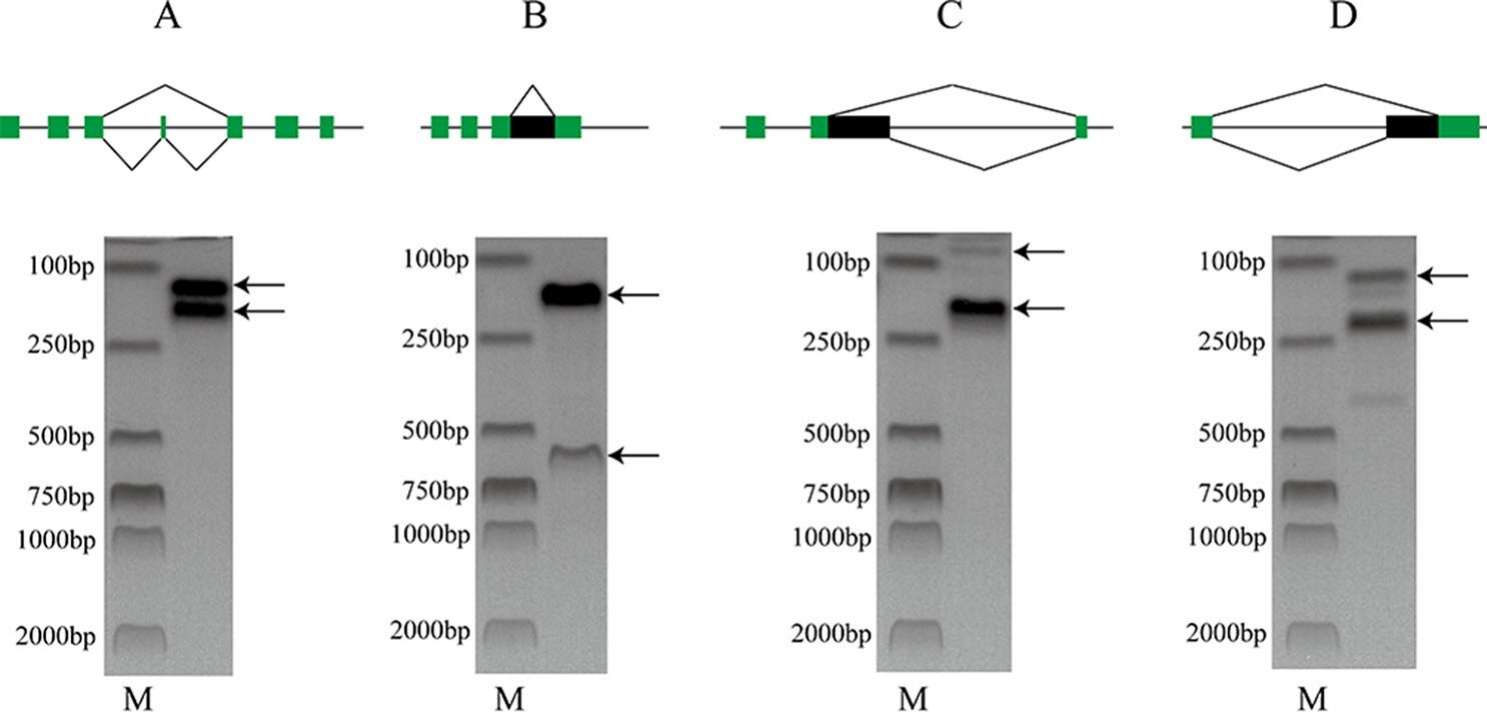
PCR validation of **(A)** ES (LVAN06071), **(B)** RI (LVAN05891), **(C)** A5SS (LVAN05891), and **(D)** A3SS (LVAN03222) events. M denotes DNA marker. The green blocks represent exons, and the black blocks represent introns.

The raw RNA-seq reads were aligned to the target genes to check the corresponding AS events (**Supplementary Figure 3**). To further test the accuracy of our prediction, ES, RI, A5SS, and A3SS events were validated by PCR (**Figure 2**). The results indicate that the AS prediction based on RNA-seq data was reliable.

### Characteristics of AS Genes

To investigate the characteristics of AS genes, we explored the gene features, gene length, exon number, exon length, and intron length of AS genes and compared with those of non-AS genes (**Table 1**). The average length of AS genes (13.0 kb) is significantly longer than that of non-AS genes (7.2 kb). The average exon number of AS genes (8.3) is significantly more than that of non-AS genes (5.1). The average exon length of AS genes (197.8 bp) is significantly shorter than that of non-AS genes (292.9 bp). The average intron length of AS genes is about 1.56 kb, which is longer than that of non-AS genes (1.39 kb).

**Table 1:** Feature comparisons between AS and non-AS genes.

Features	Gene length/kb	Exon number	Exon length/bp	Intron length/bp
AS genes	13.078*	8.322*	197.777*	1,561.310*
Non-AS genes	7.229	5.111	292.853	1,394.381

* indicates significant difference (P < 0.01) between two groups.

A totaln of 16,363 non-AS genes and 2,245 multi-AS genes with more than 10 AS isoforms were identified. The GO enrichment analysis showed that non-AS genes were mainly enriched for the terms of “DNA integration,” “DNA metabolic process,” “nucleic acid metabolic process,” “neurological system process,” and so on, it indicates that these processes are conserved in shrimp. However, multi-AS genes were mainly associated with two sets of genes. One set was enriched for GO terms “G-protein coupled receptor signaling pathway” and “signal transduction,” and the other was significantly enriched for GO terms “response to host defenses,” “response to biotic stimulus,” “response to external stimulus,” and “response to stimulus” (P < 0.00001) (**Table 2**). These terms included well-known genes like RhoGAP, Rab-3, Rab-26, Rapgef3, C-type lectin, ubiquitin, Na^+^/K^+^-transporting ATPase, Hsp40, Hsp90, nesprin-1, and so on (**Supplementary Table 2**).

**Table 2:** GO analysis and classification of biological process associated with non-AS genes and multi-AS genes (top 10).

Gene sets	ID	GO term	P value
Non-AS genes	GO:0015074	DNA integration	4.86E-12
	GO:0050909	Sensory perception of taste	7.11E-08
	GO:0003008	Neurological system process	9.79E-07
	GO:0007600	DNA metabolic process	2.01E-06
	GO:0007606	Nucleic acid metabolic process	2.83E-05
	GO:0050877	Nucleobase-containing compound metabolic process	5.45E-05
	GO:0006259	Cellular aromatic compound metabolic process	2.67E-04
	GO:0090304	Cellular nitrogen compound metabolic process	3.40E-04
	GO:0006139	Heterocycle metabolic process	5.90E-04
	GO:0006725	Sister chromatid cohesion	1.00E-03
Multi-AS genes	GO:0052200	Response to host defenses	4.10E-42
	GO:0075136	Interaction with host	9.64E-37
	GO:0051701	Response to biotic stimulus	1.41E-33
	GO:0009607	Response to external stimulus	8.06E-31
	GO:0043207	Interspecies interaction between organisms	7.61E-24
	GO:0051707	Response to stimulus	3.56E-21
	GO:0009605	Multi-organism process	3.18E-19
	GO:0044403	Chitin metabolic process	8.59E-15
	GO:0007186	G-protein-coupled receptor signaling pathway	7.01E-12
	GO:0044419	Signal transduction	1.05E-11

### AS Events in Different Stress Conditions

**Figure 3:**
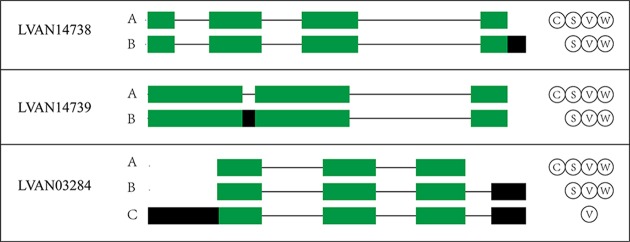
The AS patterns of three anti-lipopolysaccharide factor genes under biotic stressed conditions. The letters C, S, V, and W in the circle represent AS isoforms in the control, *S. aureus*, *V. parahaemolyticus*, and WSSV infection groups, respectively.

**Figure 4:**
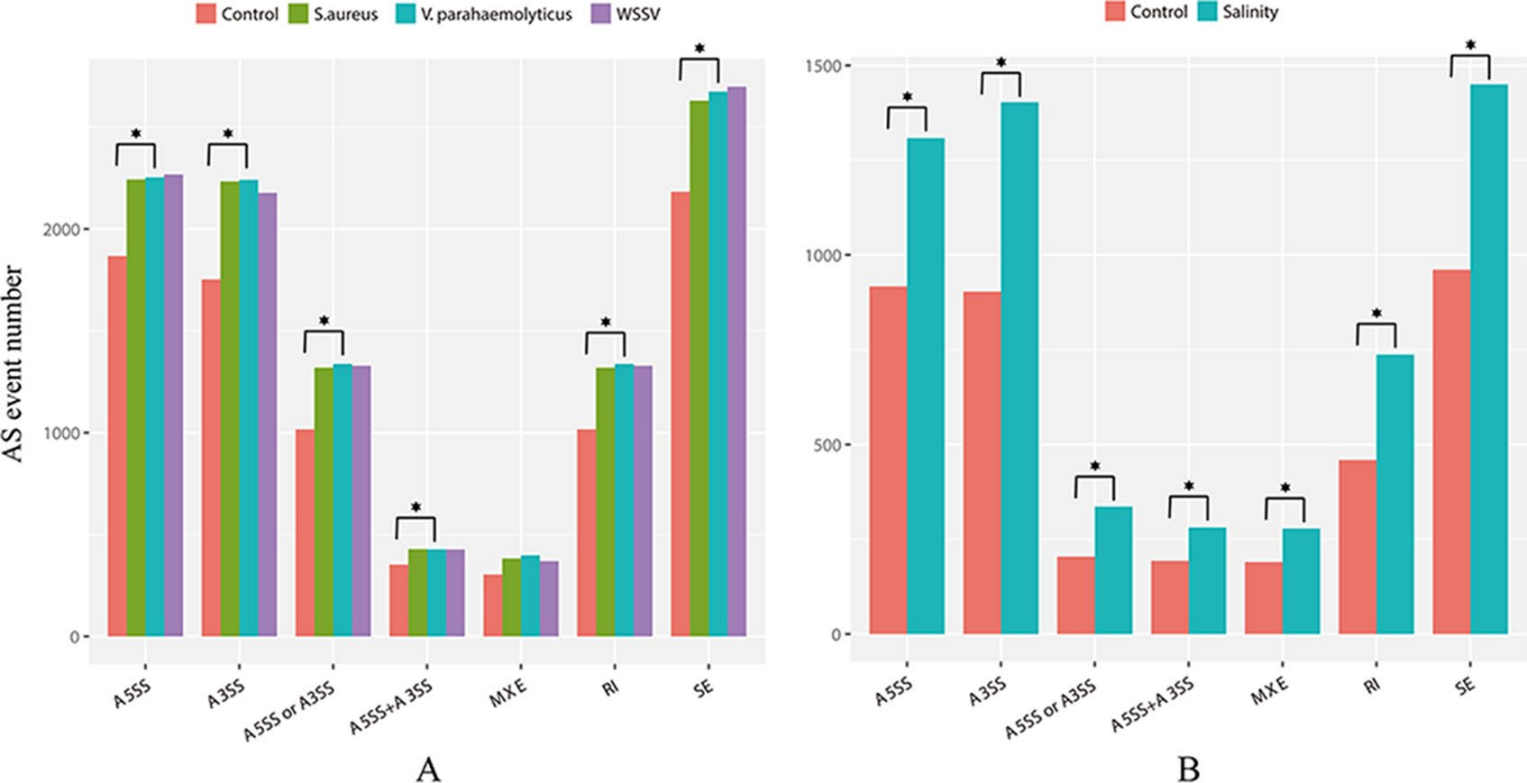
Numbers of main type of AS events in **(A)** infection libraries and **(B)** low-salinity challenged libraries. The asterisk above the column indicates a significant difference in the number of AS between the two groups.

The WSSV, *V. parahaemolyticus*, and *S. aureus* infection groups possessed 4,151, 4,310, and 4,173 AS events more than their control groups, respectively. In addition, 3,933 AS events were induced by low salinity stress. These results indicated the overall increase of the AS event number by stress. For example, the number of AS events of three anti-lipopolysaccharide factor genes increased under biotic stressed conditions (**Figure 3**). In comparison with that of control groups, AS events of ES, RI, A5SS, A3SS, A5SS, or A3SS and A5SS+A3SS were significantly higher in stressed groups (**Figure 4**). ES was increased mostly (average of 464) in stressed groups than other AS types, and A5SS or A3SS of salinity groups had the maximum increasing ratio (64.7%). However, various types of AS events were not specifically increased under stress but equally increased, suggesting that balancing selection has happened on these AS types. However, no significant differences were observed on the ratios of the main types of AS between the control groups and corresponding stressed groups (**Table 3**).

**Table 3:** Distribution of different AS types of stress libraries (top 10).

Condition	ES	A3SS	A5SS	RI	A5SS or A3SS	MXE	A5SS + A3SS	ES or A5SS	ES or A3SS	ES1 + ES2
Control (infection)	18.6%	15.0%	15.9%	8.7%	3.5%	2.6%	3.0%	1.3%	1.3%	1.3%
WSSV	17.0%	13.7%	14.3%	8.4%	3.5%	2.3%	2.7%	1.2%	1.4%	1.2%
Vibrio parahaemolyticus	16.7%	14.0%	14.1%	8.3%	3.6%	2.5%	2.7%	0.8%	1.4%	1.3%
Staphylococcus aureus	16.5%	14.1%	14.1%	8.3%	3.3%	2.4%	2.7%	1.3%	1.3%	1.2%
Control (salinity)	17.4%	16.3%	16.6%	8.3%	3.7%	3.4%	3.5%	2.0%	1.6%	1.4%
Salinity	15.3%	14.8%	13.8%	7.8%	3.6%	2.9%	3.0%	1.9%	1.8%	1.2%

### Splicing Site Motif Patterns

**Figure 5:**
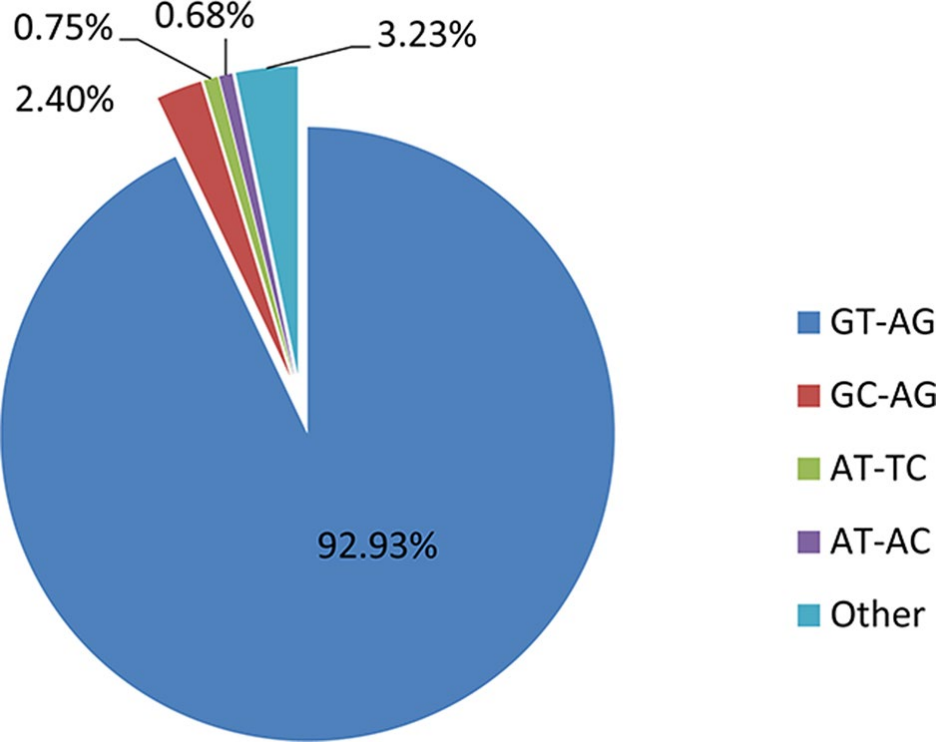
Proportion of different splicing usages of all genome-predicted genes.

In the shrimp genome, GT-AG, GC-AG, AT-TC, and AT-AC were the main four types of splice site, which was consistent with those of human, fruitfly, and oyster (Thanaraj and Clark, 2001; Graveley et al., 2011; Huang et al., 2016). Among them, 92.93% use the canonical GT-AG dinucleotides at the 5’ and 3’ sites, followed by GC-AG (2.4%) and AT-TC (0.75%) (**Figure 5**). When investigating whether non-AS genes and AS genes possess different splicing sites motif patterns, the result showed that similar to the whole genome predicted genes, more than 95% splicing sites of both AS genes and non-AS genes were GT-AG dinucleotide (**Tables 4** and **5**). However, no significant difference was observed between stressed groups and corresponding control groups.

**Table 4:** Splicing sites motif of all non-AS transcripts of each library.

Condition	GT-AG	GC-AG	AT-TC	AT-AC	NN-AG	Other
Control (infection)	97.82%	1.04%	0.18%	0.19%	0.05%	0.72%
WSSV	97.96%	1.00%	0.19%	0.18%	0.03%	0.64%
*Vibrio parahaemolyticus*	97.93%	1.03%	0.19%	0.19%	0.03%	0.63%
*Staphylococcus aureus*	97.88%	1.04%	0.18%	0.19%	0.04%	0.67%
Control (salinity)	98.73%	0.79%	0.07%	0.06%	0.02%	0.33%
Salinity	98.40%	0.87%	0.10%	0.08%	0.03%	0.52%

“NN” indicates repeat sequences of shrimp genome.

**Table 5:** Splicing sites motif of all AS transcripts of each library.

Condition	GT-AG	GC-AG	AT-TC	AT-AC	NN-AG	Other
Control (infection)	95.72%	1.48%	0.19%	0.24%	0.17%	2.21%
WSSV	95.73%	1.42%	0.18%	0.20%	0.16%	2.32%
*Vibrio parahaemolyticus*	95.54%	1.48%	0.15%	0.24%	0.17%	2.42%
*Staphylococcus aureus*	95.57%	1.49%	0.19%	0.24%	0.16%	2.36%
Control (salinity)	96.03%	1.28%	0.16%	0.15%	0.16%	2.21%
Salinity	95.56%	1.41%	0.18%	0.16%	0.15%	2.54%

“NN” indicates repeat sequences of shrimp genome.

### Type 1 AS Genes of Stressed Libraries

**Figure 6:**
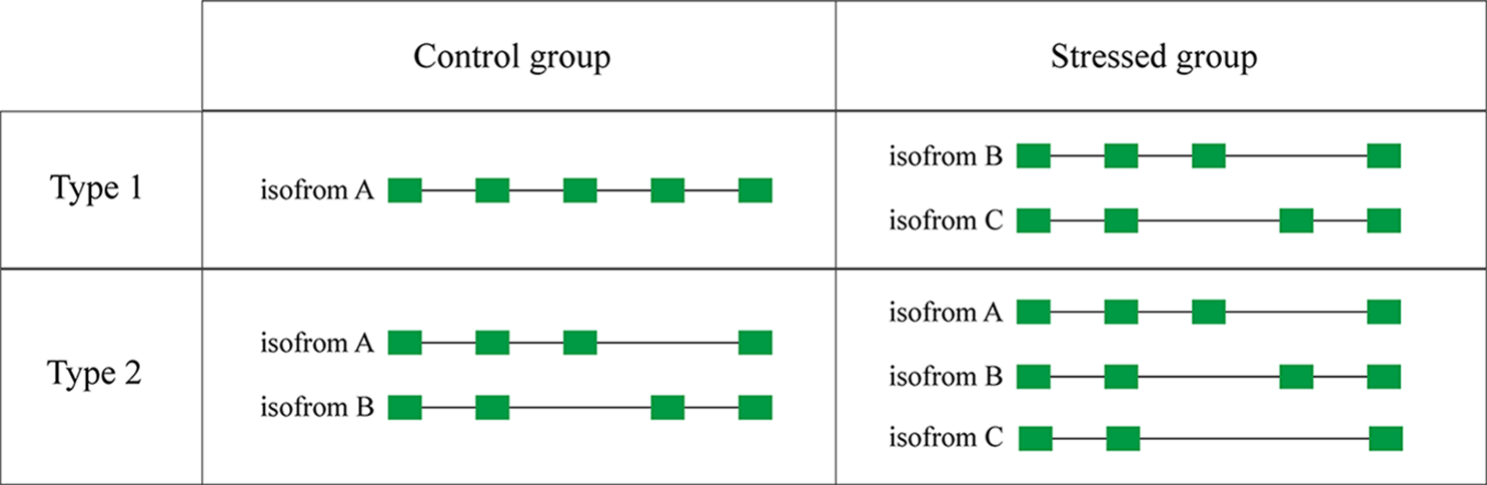
Schematic diagram of type 1 AS genes and type 2 AS genes.

Some genes possess only one transcript in the control group but were alternatively spliced in the experimental group of stressed libraries. These genes were defined as type 1AS genes (**Figure 6**). There are 854, 855, 851, and 1,002 type 1 AS genes identified under WSSV infection, *V. parahaemolyticus* infection, *S. aureus* infection, and salinity response, respectively (**Table 6**).

**Table 6:** The number of type 1 AS genes and type 2 AS genes under different stress conditions.

	AS event number	Related gene number	Type 1 AS gene number	Type 2 AS gene number
Control (infection)	11,717	4,890	/	/
WSSV	15,868	5,649	854	2,515
*Vibrio parahaemolyticus*	16,027	5,666	855	2,507
*Staphylococcus aureus*	15,890	5,658	851	2,434
Control (salinity)	5,524	2,818	/	/
Salinity	9,457	3,762	1002	2,090

**Figure 7:**
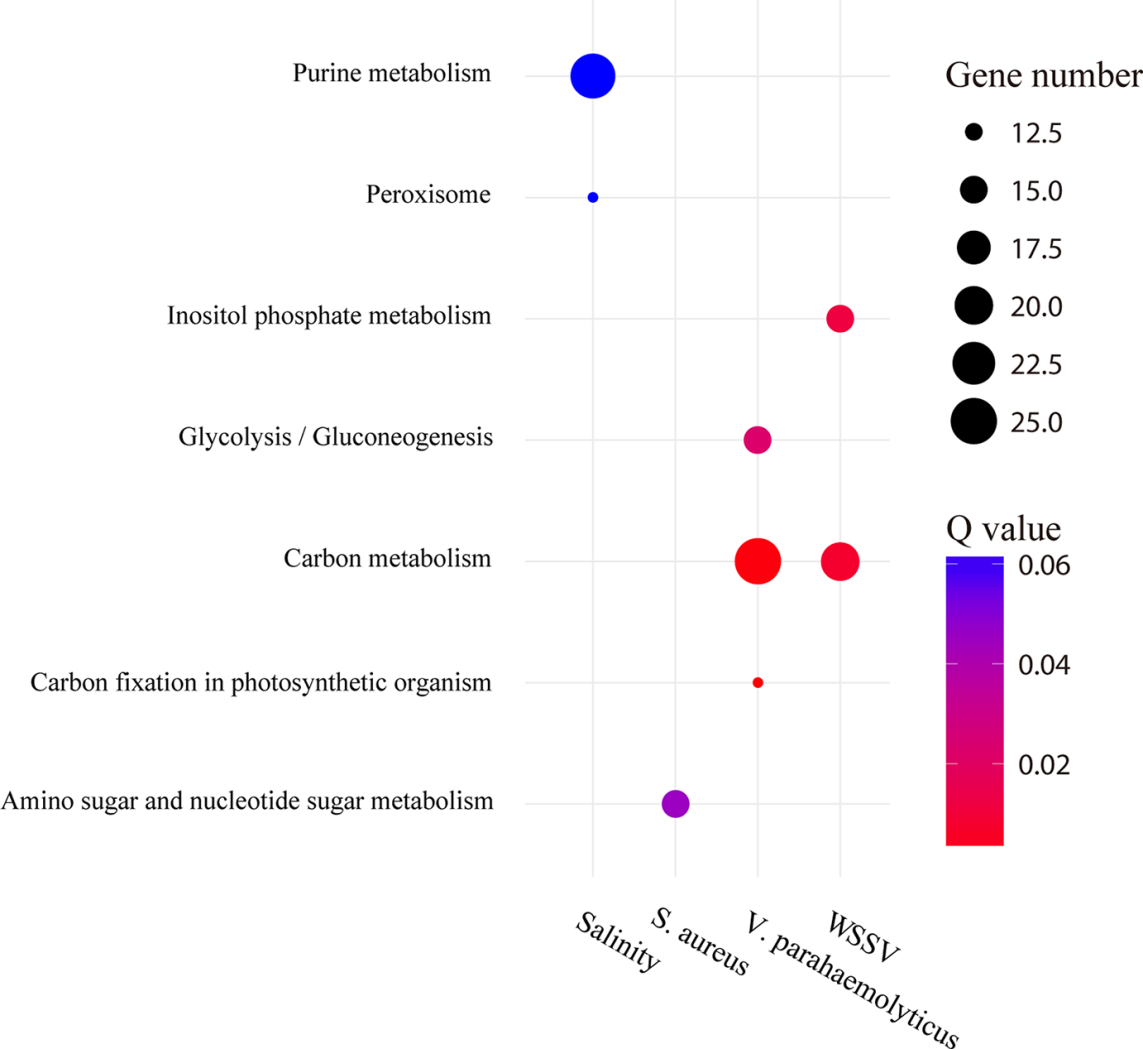
Pathway enrichment analysis of type 1 AS genes under stress conditions. The circle size and filled portions represent the gene numbers of type 1 AS gene in a given pathway. The statistical significance is colored according to Q values.

GO enrichment analysis was performed on type 1 AS genes (**Supplementary Figure 4**). The results showed that they were mainly associated with substance metabolism or energy metabolism. For instance, the type 1 AS genes of WSSV and *S. aureus* library were mainly enriched in “amino sugar metabolic process” (GO:0006040), “aminoglycan metabolic process” (GO:0006022), and “glucosamine-containing compound metabolic process” (GO:1901071) for biological processes. For molecular function, the type 1 AS genes of WSSV and *V. parahaemolyticus* library were predominantly enriched in “triose-phosphate isomerase activity” (GO:0004807) and “interconverting aldoses and ketoses” (GO:0016861). The KEGG enrichment analysis indicated type 1 AS genes enriched in the terms “carbon metabolism” (ko01200) after WSSV and *S*. aureus infection. In addition, we found the type 1 AS genes after *V. parahaemolyticus* infection enriched for the term “amino sugar and nucleotide sugar metabolism” (ko00520) (**Figure 7**). Two triose-phosphate isomerase genes were selected to illustrate the effect of AS on the protein structures (**Supplementary Figure 5**). Furthermore, 203 type 1 AS genes were common in WSSV, *V. parahaemolyticus*, and *S. aureus* library (**Supplementary Figure 6**), it indicated that many common biological processes have changed as a result of these three infections. As for salinity stressed library, the type 1 AS genes showed a degree of enrichment for the term “peroxisome” (ko04146) and “purine metabolism” (ko00230) (**Figure 7**).

### Type 2 AS Genes of Stressed Libraries

Compared with the control group, some genes that possessed specific AS events in the experimental group under stressed conditions were considered as type 2 AS genes (**Figure 6**). In total, 6,488, 6,653, 6,543, and 5,217 type 2 AS events were identified after WSSV infection, *V. parahaemolyticus* infection, *S. aureus* infection, and salinity response, respectively (**Table 6**), corresponding to 2,515, 2,507, 2,434, and 2,090 genes.

**Figure 8:**
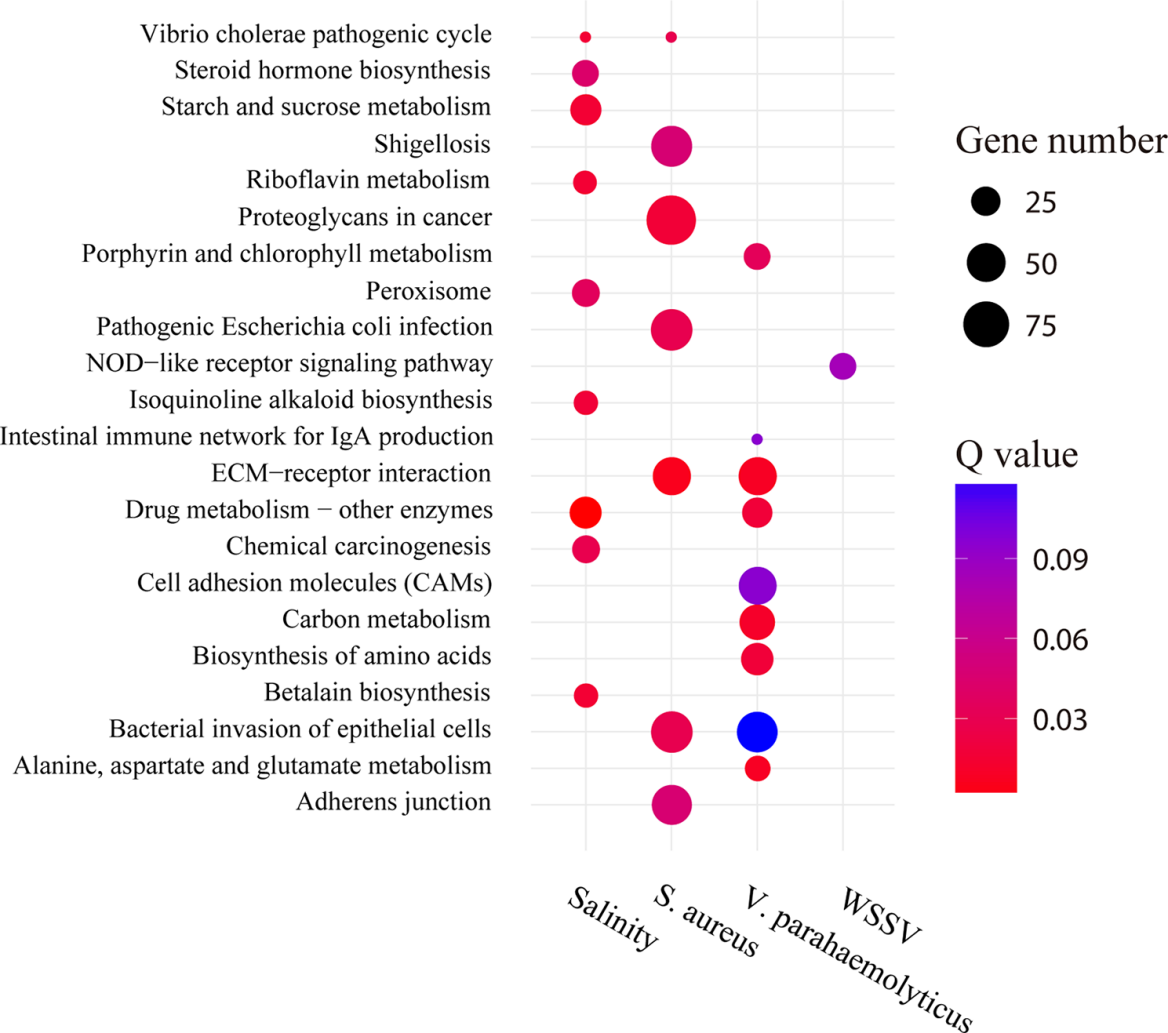
Pathway enrichment analysis of type 2 AS genes under stress conditions. The circle size and filled portions represent the gene numbers of type 2 AS genes in a given pathway. The statistical significance is colored according to Q values.

GO enrichment analysis showed that the proportions of enriched GO terms for type 2 AS genes were globally similar based on molecular function and cellular component (**Supplementary Figure 7**). For cellular components, type 2 AS genes were predominantly enriched in GO terms “cell” (GO:0005623), “cell part” (GO:0044464), and so on. For the molecular function category, the majority of type 2 AS genes were annotated with the term “binding” (GO:0005488) and so on. However, the KEGG enrichment analysis result of these genes was relatively specific (**Figure 8**). For example, the type 2 AS genes showed a degree of enrichment for the term “NOD-like receptor signaling pathway” (ko04621) after WSSV infection and including well-known genes (such as ERK and TAK1). In addition, the type 2 AS genes enriched for the term “ECM receptor interaction” (ko04512) and “intestinal immune network for IgA production” (ko04672) after *V. parahaemolyticus* infection. The former pathway includes integrin gene, and the latter includes integrin-related genes. As for salinity stressed library, “betalain biosynthesis” (ko00965) was the most enriched term and includes hemocyanin and prophenoloxidase genes.

### Alteration of Splicing Factors in AS Patterns

**Figure 9:**
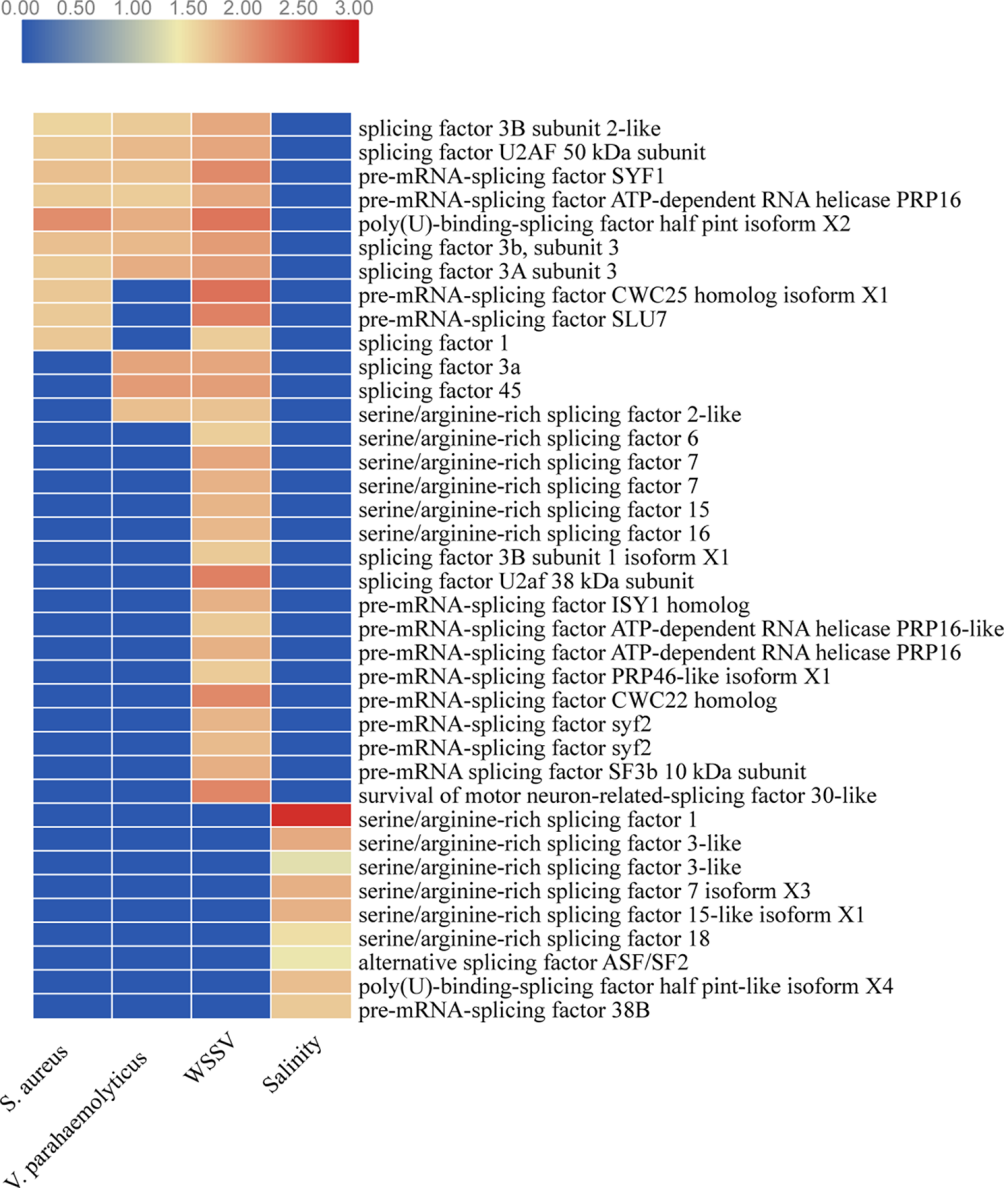
The expression levels of splicing factors under stressed conditions. Red color represents significantly upregulated genes. Blue color represents genes with no significant expression.

Splicing factors are functionally essential in AS events that can alternatively splice pre-mRNA by preferentially selecting different splice sites and generate multiple mRNA transcripts from one pre-mRNA transcript, such as SR (serine/arginine-rich) splicing factor and U2AF (Hsin-Chou and Soo-Chen, 2012). Strikingly, the expression 10, 10, 29, and 9 splicing factor genes was significantly upregulated under stressed conditions for WSSV infection, *V. parahaemolyticus* infection, *S. aureus* infection, and salinity response, respectively (**Figure 9**). These splicing factors might contribute to the overall promotion of AS number and play key roles in the execution and regulation of pre-mRNA splicing in shrimp under stress conditions.

Similar splicing factors seem to regulate the pre-mRNA splicing in shrimp under biotic stress conditions. For example, the expression levels of seven splicing factors (splicing factor 3B subunit 2-like, splicing factor U2AF 50 kDa subunit, pre-mRNA-splicing factor SYF1, pre-mRNA-splicing factor ATP-dependent RNA helicase PRP16, poly(U)-binding-splicing factor half pint isoform X2, splicing factor 3b subunit 3, and splicing factor 3A subunit 3) were significantly upregulated under biotic stressed conditions. However, the splicing factors that were expressed differently were distinct under abiotic stress conditions (**Figure 9**). These results indicate that splicing factors might respond to stressed conditions specifically and thus benefit shrimp, adapting to various environmental stressed conditions.

## Discussion

In this study, 38,781 AS events corresponding to 39.5% of the multi-exon genes indicate the ubiquity of AS genes in the shrimp genome, like other invertebrates. ES is the most abundant AS type, followed by A3SS, A5SS, RI, A5SS/A3SS, and MXE. This distribution pattern is consistent with that of other animals reported previously (Pan et al., 2008; Wang et al., 2008; Lu et al., 2010; Graveley et al., 2011; Ramani et al., 2011; Huang et al., 2016). Comprehensively, these results suggest that animals might possess similar AS splice forms.

The response to any given stress is a sophisticated process in animals, which involved many biological mechanisms interpenetrate and interact, including the regulation of gene expression at transcriptional and posttranslational levels. Alternative splicing has been proposed as one of the regulatory mechanisms that promote genome plasticity and versatility. Under normal circumstances, non-AS genes tend to participate in the conserved biological processes in shrimp, such as DNA replication and nucleic acid metabolic process. However, multi-AS genes are mainly composed of stress-responsive genes in shrimp. It seems that multi-AS stress-responsive genes may cope with various stressed conditions.

But what will happen when stressed conditions really come? How do stressed conditions affect global AS patterns of shrimp? First, no significant changes of the alternative splice motif patterns were observed under stressed conditions, suggesting that selection pressure does not act on splice sites motif. Similar results were found in the Pacific oyster under salinity, temperature-treated, and air exposure conditions (Huang et al., 2016). Second, AS events were obviously increased in shrimp under biotic and abiotic stresses, which were consistent with those of plants under salt stress (Iida et al., 2004; Feng et al., 2014) and temperature stress (Mastrangelo et al., 2005). Third, AS event number of the main type increases overall under stress. Further functional analysis showed that the type 1 AS genes were mainly related with material metabolism and energy metabolism pathways, such as amino sugar metabolism and carbon metabolism under biotic and abiotic stress experiments, whereas the type 2 AS genes were involved in different response pathways under different stress conditions. These results suggest that the promotion of AS events is not a random process but associated with the different stress responses. For instance, the type 2 AS genes showed a degree of enrichment for the term “NOD-like receptor signaling pathway” after WSSV infection. NOD-like receptor signaling pathway includes many families of pattern recognition receptors, driving the activation of NF-κB and MAPK, cytokine production, and apoptosis, responsible for detecting various pathogens and generating innate immune responses (Kanneganti et al., 2007). The “betalain biosynthesis” is the most enriched term of type 2 AS genes in low-salinity stressed library. It is a pivotal pathway response to osmotic adjustment and salt resistance. As a ubiquitous secondary metabolite of metabolism, betaine is very important to enhance stress resistance, such as drought and salinity resistance (Hayakawa and Agarie, 2010; Jain and Gould, 2015).

On the basis of these results, we can infer that when subjected to stress conditions, shrimp will activate the alternative splice of genes related to material metabolism to prevent the disorder of basal metabolism, and the genes associated with energy metabolism will also be alternatively spliced to ensure enough substrate and energy supply. At the same time, alternative splicing pattern of the stress-responsive genes will also be changed to alter their own expression levels or function and regulate other genes in turn, then considerably enhance and amplify the signal transduction cascade in response to the stress condition. This may be an effective approach for shrimp to respond to stress conditions efficiently. This approach is originally started by splicing factors, but more evidence is needed to know how splicing factors accurately regulate the global AS patterns.

## Conclusion

In this study, we first disclosed features of genome-wide AS in shrimp under numerous stresses through comprehensive transcriptome analysis of the shrimp high-throughput RNA-seq data. The results suggest that 39.5% of the intron-containing genes in shrimp genome are alternatively spliced. Moreover, we found that the AS number is increased under stressed conditions. The analysis of functional categories demonstrates that type 1 AS genes under stressed conditions are associated with substance and energy metabolism, and the type 2 AS genes under stressed conditions have different responses to corresponding stresses. Our analysis provides the most comprehensive information on alternatively spliced transcripts in shrimp to date and will be of great value in addressing regulation of expression and gene/protein function.

## Data Availability

Publicly available datasets were analyzed in this study. This data can be found here: SRR1460493, SRR1460494, SRR1460495, SRR1460504, SRR1460505, SRX1098368, SRX1098369, SRX1098370, SRX1098371, SRX1098372, SRX1098373, SRX1098374, SRX1098375, SRX3886087, SRX3886088, SRX3886089, SRX3886090, SRX3556303, SRX3556304, SRX3556305, SRX3556306, SRX3556307, SRX3556308, SRX3556309, SRX3556310, SRX3556311, SRX3556312, SRX3556291, SRX3556292, SRX3556278, SRX3556279, SRX3556280, SRX3556257.

## Ethics Statement

This study was carried out in accordance with the recommendations of Welfare ethics of experimental animals and safety inspection system of animal experiments, laboratory animal management and ethics Committee of IOCAS. The protocol was approved by the laboratory animal management and ethics Committee of IOCAS.

## Author Contributions

JY, CL and XiaoxZ conceived and designed the study. XiaoxZ collected the data, conducted the bioinformatics analyses and wrote the manuscript. XiaojZ, JY, JX and FL revised the manuscript. All authors read and approved the final manuscript.

## Funding

This work was financially supported by the National Natural Science Foundation of China (31830100, 41776158), the National Key R&D Program of China (2018YFD0900103) and the China Agriculture Research System-48 (CARS-48).

## Conflict of Interest Statement

The authors declare that the research was conducted in the absence of any commercial or financial relationships that could be construed as a potential conflict of interest.

The reviewer KJ declared a shared affiliation, though no other collaboration, with the authors to the handling Editor.
