# Supplementary material for: Genome-Wide Analysis of Alternative Splicing Provides Insights Into Stress Response of the Pacific White Shrimp Litopenaeus vanname
Source: Front Genet. 2019 Sep 12;10:845. doi: 10.3389/fgene.2019.00845 (PMC6752684; doi:10.3389/fgene.2019.00845)
Supplement: Supplementary file 1 [file DataSheet_1.doc]

**Supplementary table 1.** The RT-PCR primers used for AS validation.

| Primers | Sequences (5’-3’) |
| --- | --- |
| LVAN05891-A5SS-F2 | GCGAAGAACAACGACTGGTC |
| LVAN05891-A5SS-R2 | TTCCTCCTGCTGGTGATGTAG |
| LVAN05891-RI-F2 | GGCGTTGCTTCTCCGAGTA |
| LVAN05891-RI-R2 | CCTGCTCCTTCTGTGTTCTCT |
| LVAN06071-ES-F | CCACATACAGGTTTCAGGTCTTT |
| LVAN06071-ES-R | GTGCTCTTCACACTGGTAACAT |
| LVAN03222-A3SS-F1 | TCTTGGAGCCGAGCACCTA |
| LVAN03222-A3SS-R1 | GCCAGGGACTTGAGCTTGAT |

**Supplementary table 2.** List of some representative multi-AS genes associated with response to stimulus and signaling pathway.

| Annotation | Function | Annotation | Function |
| --- | --- | --- | --- |
| Crustacyanin subunit A | Response to stimulus | PLCE1 | Response to stimulus |
| Crustacyanin subunit C | Response to stimulus | Regulator of G-protein signaling 7 | Response to stimulus |
| Myosin heavy chain | Response to stimulus | Rho GTPase-activating protein | GPCR signaling pathway |
| Rho GTPase-activating protein 20 | Response to stimulus | SH3 and cysteine-rich domain-containing protein 2 | GPCR signaling pathway |
| Polyubiquitin-A | Response to stimulus | Thrombospondin-3b | GPCR signaling pathway |
| Rab-3 | Response to stimulus | ELAV-like protein 4 | GPCR signaling pathway |
| Rab-26 | Response to stimulus | Peritrophin-1 | Signal transduction |
| Nesprin-1 | Response to stimulus | Ankyrin-3 | GPCR signaling pathway |
| Sodium/potassium-transporting ATPase | Response to stimulus | Rap guanine nucleotide exchange factor 1 | GPCR signaling pathway |
| Rap guanine nucleotide exchange factor 2 | Response to stimulus | GTP-binding protein Rheb homolog | GPCR signaling pathway |
| Heat shock protein 40 | Response to stimulus | Ca(2+)/calmodulin-responsive adenylate cyclase | Intracellular signal transduction |
| Heat shock protein 90 | Response to stimulus | Fibropellin-1 | GPCR signaling pathway |
| C-type lectin | Response to stimulus | Adenomatous polyposis coli protein | Wnt receptor signaling pathway |
| Protein kinase C | Response to stimulus | Furin-like protease 2 | transmembrane receptor protein tyrosine kinase signaling pathway |
| SE-cephalotoxin | Response to stimulus | Leucine-rich repeat serine/threonine-protein kinase 1 | small GTPase mediated signal transduction |
| Diacylglycerol kinase | Response to stimulus | ANK repeat and PH domain-containing protein 1 | intracellular signal transduction |
