# Supplementary material for: Genome-Wide Analysis of Alternative Splicing Provides Insights Into Stress Response of the Pacific White Shrimp Litopenaeus vanname
Source: Front Genet. 2019 Sep 12;10:845. doi: 10.3389/fgene.2019.00845 (PMC6752684; doi:10.3389/fgene.2019.00845)
Supplement: Supplementary file 2 [file Presentation_1.pdf]

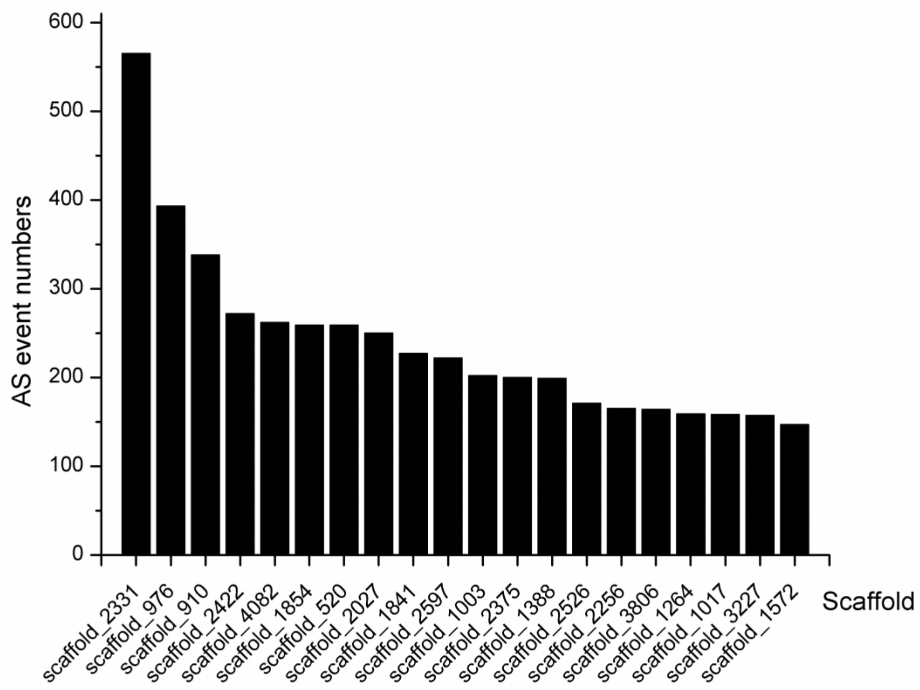

Supplementary Figure 1. AS events distribution among top 20 scaffolds.

Type	Structure	Events	Frequency (%)
A5SS1 or A5SS2 or A5SS3	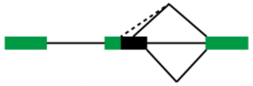	298	0.77
A3SS1 or A3SS2 or A3SS3	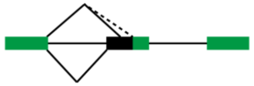	280	0.72
ES or A3SS	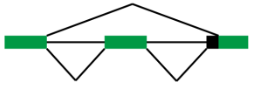	262	0.68
ES with 2 acceptors	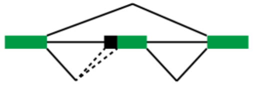	235	0.61
ES with 2 donors	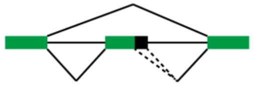	234	0.60
ES or A5SS	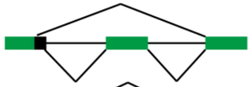	231	0.60
ES1+ES2+ES3	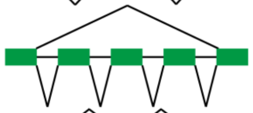	169	0.44
IR1 or IR2 or IR3	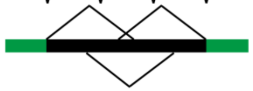	150	0.39
Other		15021	38.73

Supplementary Figure 2. Complex AS types in shrimp.

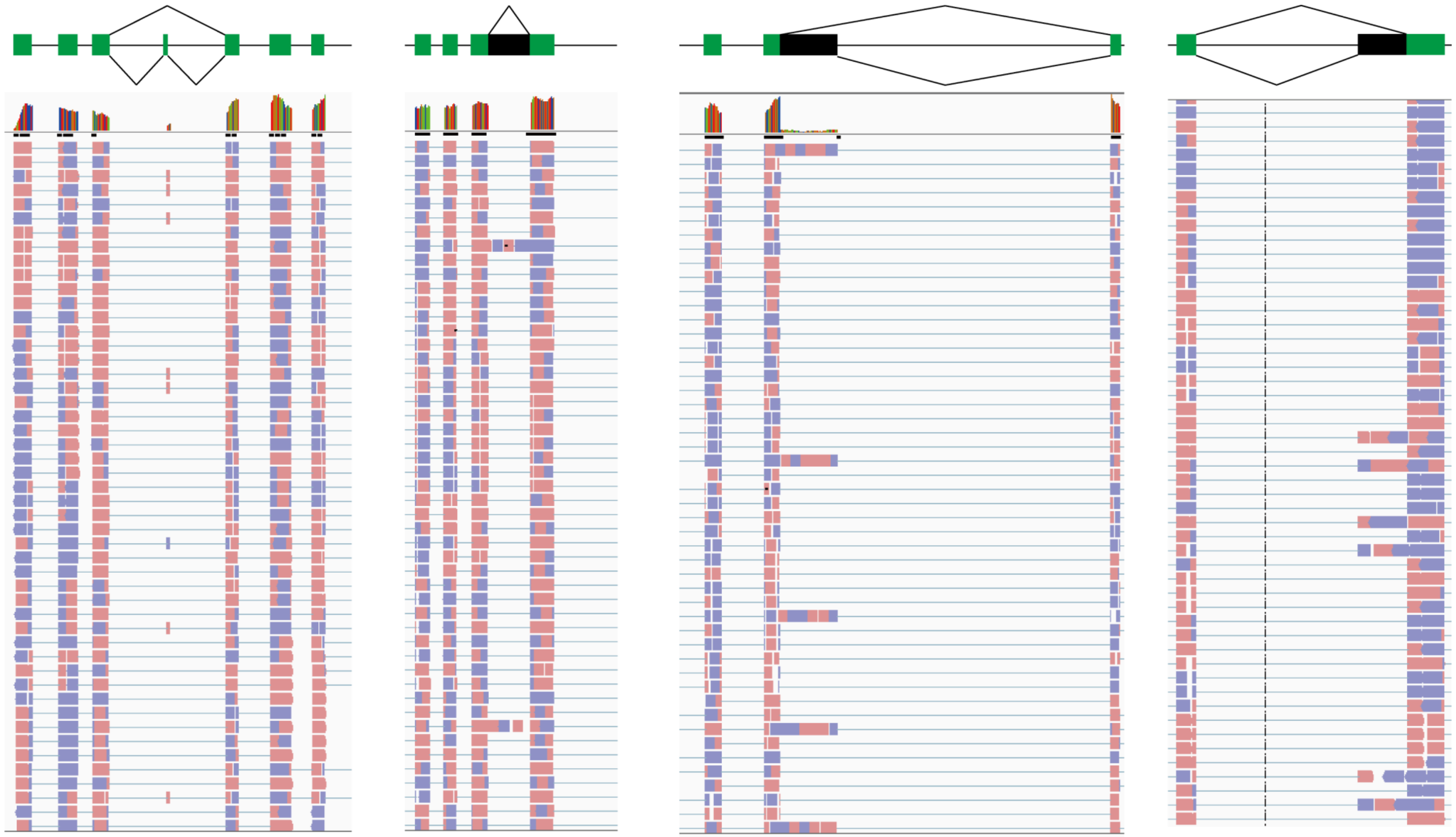

Supplementary Figure 3A. Reads distribution of prevalent AS types. From left to right: ES in LVAN06071, RI in LVAN06540, A5SS in LVAN05891, A3SS in LVAN05801. The upper shows the structure of AS events and the lower shows the mapped reads.

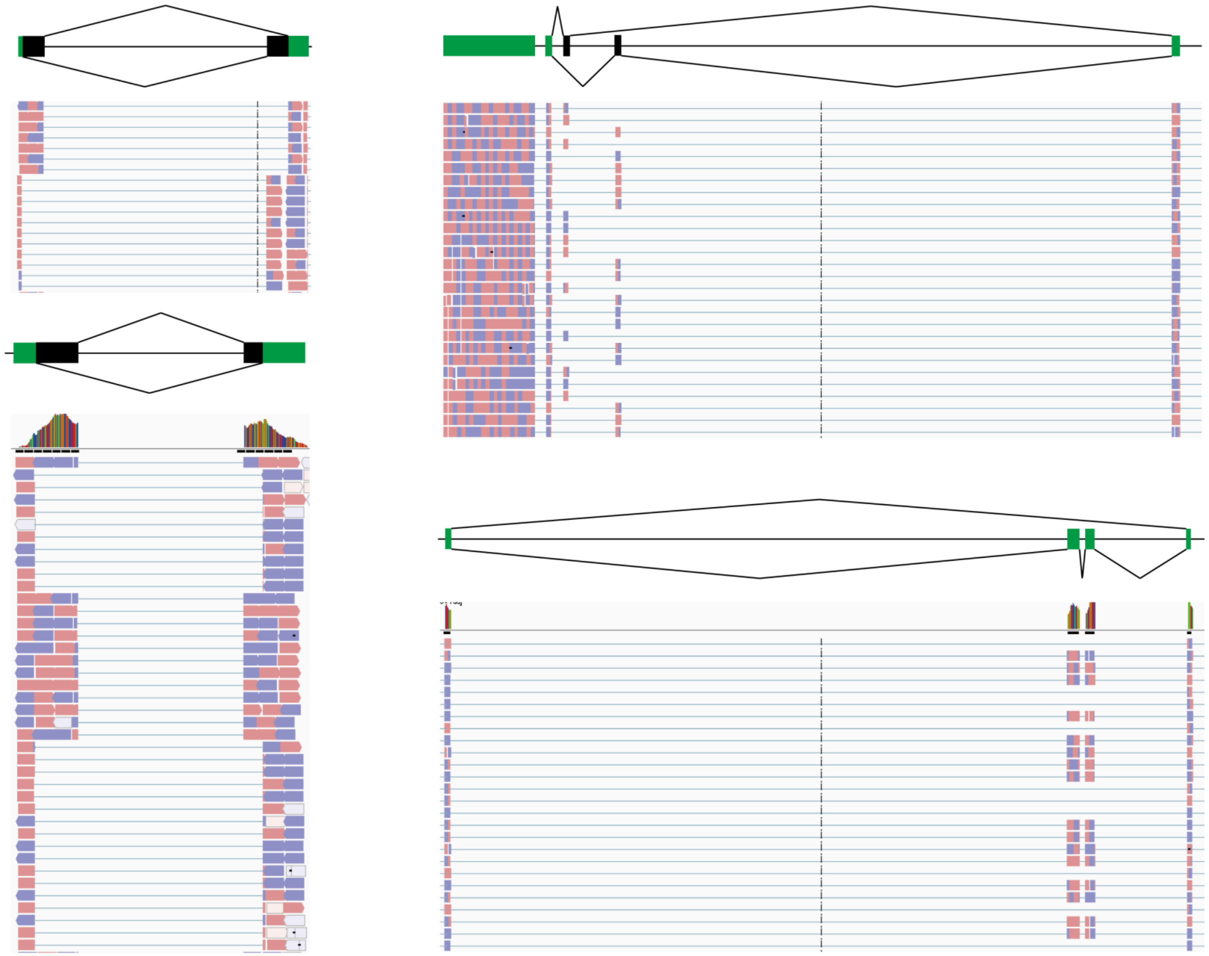

Supplementary Figure 3B. Reads distribution of prevalent AS types. From left to right andtop to bottom: A5SS or A3SS in LVAN13525, A5SS + A3SS in LVAN05137, MXE in LVAN03745, ES1+ES2 in LVAN03215. The upper shows the structure of AS events and the lower shows the mapped reads.

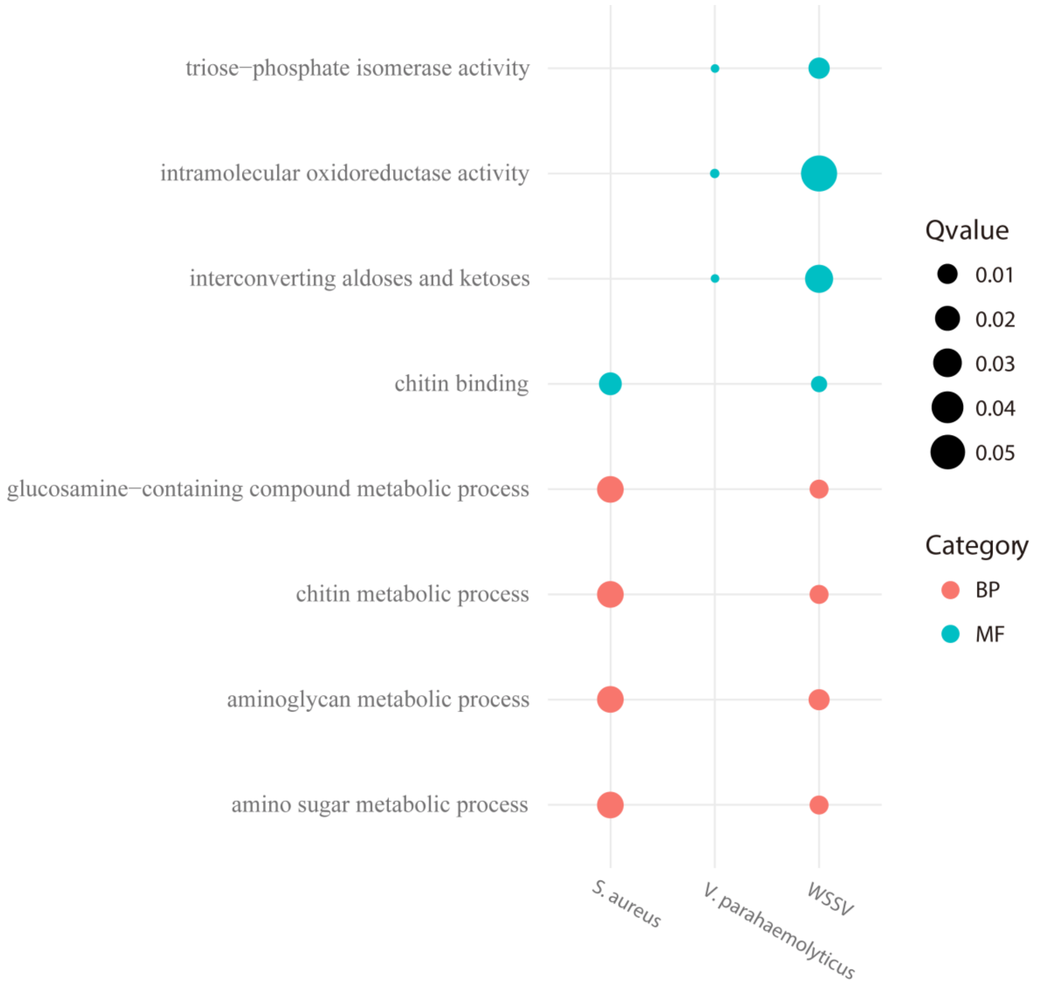

Supplementary Figure 4. The enriched GO terms of type 1 AS genes under different stressed condition. No significant enriched GO terms were found in low salinity stressed and ammonia-exposure libraries.

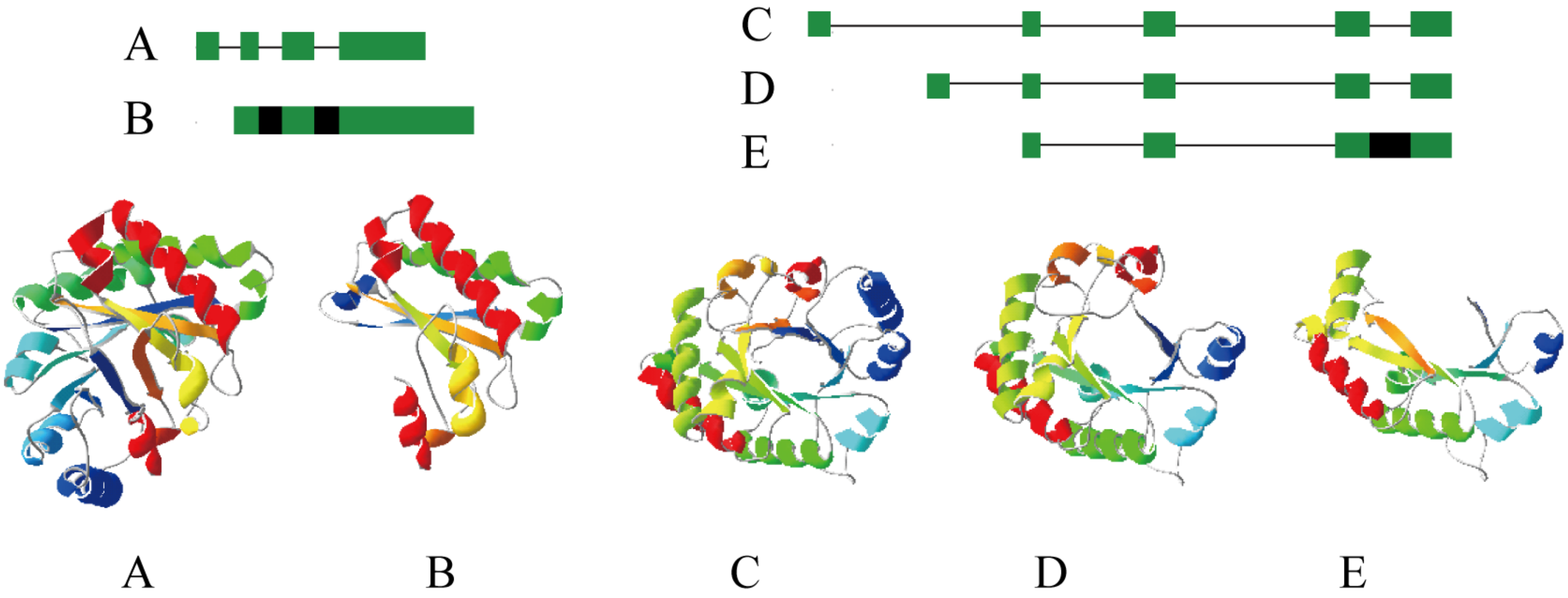

Supplementary Figure 5. The AS events of two triose phosphate isomerase (TPI) genes and their corresponding 3D structure. (A-B) represent the gene structure of LVAN03817 in control and *V. parahaemolyticus* library, respectively. (C-E) represent the gene structure of LVAN18205 in control and WSSV library, respectively.

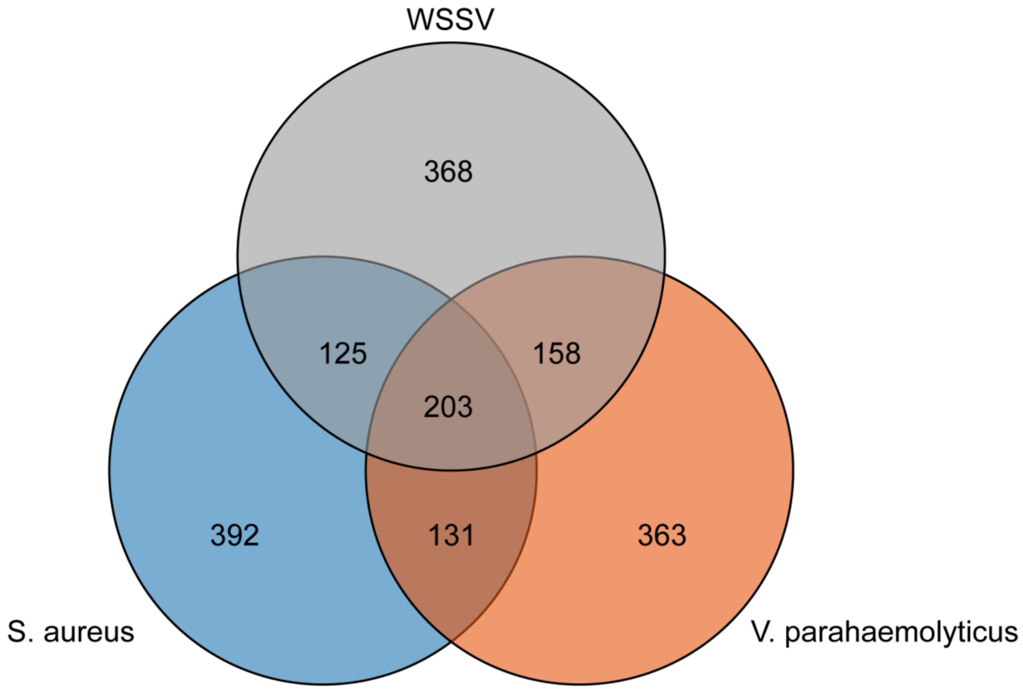

Supplementary Figure 6. Venn diagram of class 1 AS genes in WSSV, *V. parahaemolyticus* and *S. aureus* libraries.

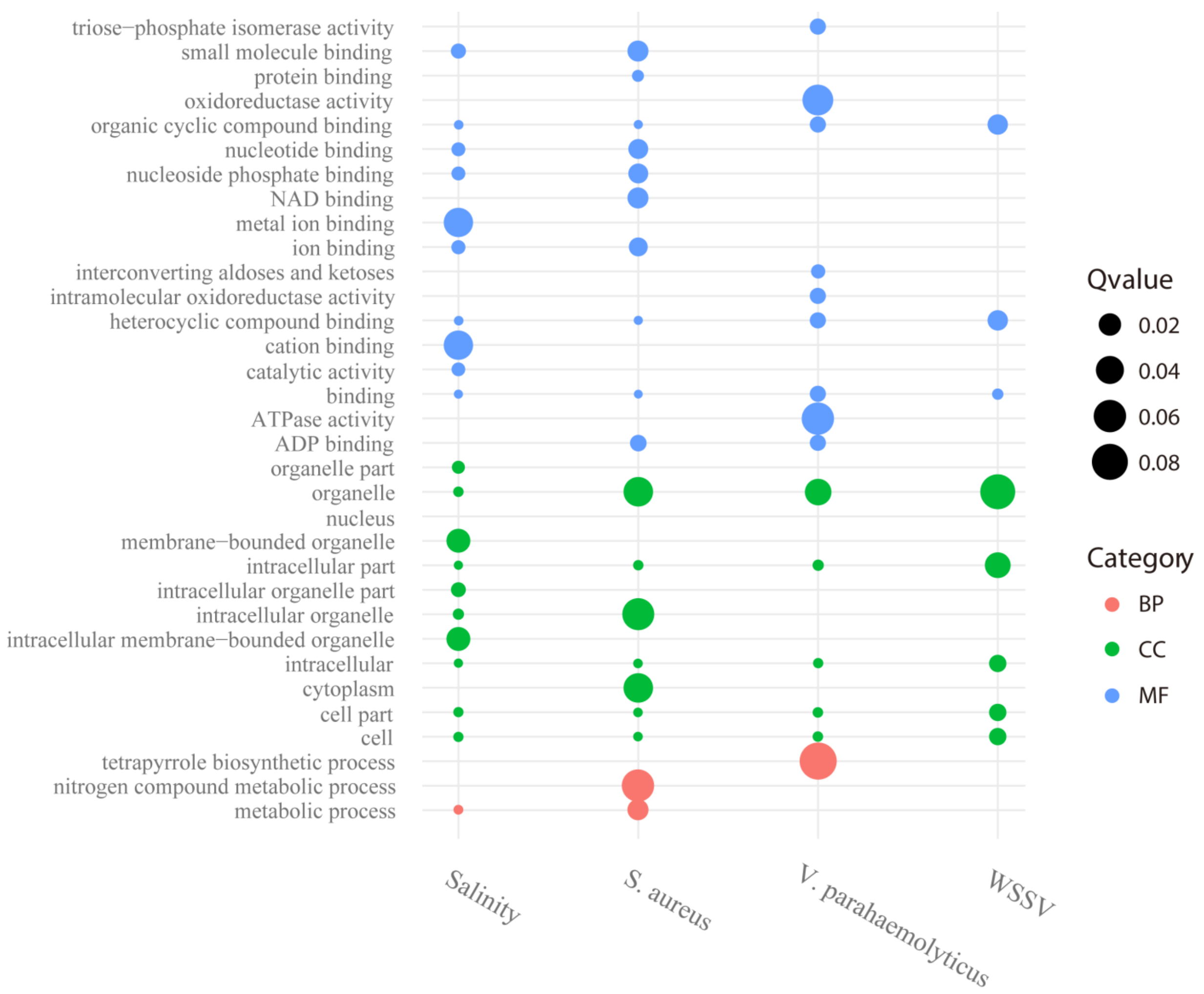

Supplementary Figure 7. The enriched GO terms of type 2 AS genes under different stressed condition.
